# Mid-upper arm circumference cut-offs for screening thinness and severe thinness in Indian adolescent girls aged 10–19 years in field settings

**DOI:** 10.1017/S1368980019000594

**Published:** 2019-08

**Authors:** Vani Sethi, Neha Gupta, Sarang Pedgaonkar, Abhishek Saraswat, Konsam Dinachandra Singh, Hifz Ur Rahman, Arjan de Wagt, Sayeed Unisa

**Affiliations:** 1 UNICEF, India Country Office, UNICEF House, 73 Lodi Estate, New Delhi – 110002, India; 2 Department of Mathematical Demography and Statistics, International Institute of Population Sciences, Mumbai, India

**Keywords:** Anthropometry, BMI, Undernutrition, Mid-upper arm circumference, Adolescent

## Abstract

**Objective:**

(i) To assess diagnostic accuracy of mid-upper arm circumference (MUAC) for screening thinness and severe thinness in Indian adolescent girls aged 10–14 and 15–19 years compared with BMI-for-age *Z*-score (BAZ) <−2 and <−3 as the gold standard and (ii) to identify appropriate MUAC cut-offs for screening thinness and severe thinness in Indian girls aged 10–14 and 15–19 years.

**Design:**

Cross-sectional, conducted October 2016–April 2017.

**Setting:**

Four tribal blocks of two eastern India states, Chhattisgarh and Odisha.

**Participants:**

Girls (*n* 4628) aged 10–19 years. Measurements included height, weight and MUAC to calculate BAZ. Standard diagnostic accuracy tests, receiver–operating characteristic curves and Youden index helped arrive at MUAC cut-offs at BAZ < −2 and <−3, as gold standard.

**Results:**

Mean MUAC and BMI correlation was positive (0·78, *P* = 0·001 and *r* ^2^ = 0·61). Among 10–14 years, MUAC cut-off corresponding to BAZ < −2 and BAZ < −3 was ≤19·4 and ≤18·9 cm. Among 15–19 years, corresponding values were ≤21·6 and ≤20·7 cm. For both BAZ < −2 and BAZ < −3, specificity was higher in 15–19 *v*. 10–14 years. State-wise variations existed. MUAC cut-offs ranged from 17·7 cm (10 years) to 22·5 cm (19 years) for BAZ < −2, and from 17·0 cm (10 years) to 21·5 cm (19 years) for BAZ < −3. Single-age area under the curve range was 0·82–0·97.

**Conclusions:**

Study provides a case for use of year-wise and sex-wise context-specific MUAC-cut-offs for screening thinness/severe thinness in adolescents, rather than one MUAC cut-off across 10–19 years, depending on purpose and logistic constraints.

The WHO recommends a BMI-for-age *Z*-score (BAZ) of <−2 and <−3, respectively, for classifying thinness and severe thinness in adolescents aged 10–19 years^(^^1^^)^. In field settings which are remote or where availability of resources (skilled manpower and financial resources) is a challenge, mid-upper arm circumference (MUAC) has also been used as a field-friendly alternative for screening thinness in adolescents (Table 1)^(^^2^^–^^9^^)^.

**Table 1:** Sample country-specific mid-upper arm circumference (MUAC) cut-offs for adolescents for screening severe thinness

Author, year	Country	Age (years)	MUAC cut-off (cm)	Results	Reference
WHO, 2011	–	–	<16·0	Admission criteria for therapeutic feeding	2
National CMAM guidelines, Sudan, 2017	Sudan, South Sudan	10–18	<16·0	Admission criteria for therapeutic feeding	3
National CMAM guidelines, Somalia, 2010	Somalia	10–18	<16·0	Admission criteria for therapeutic feeding	4
National CMAM guidelines, Ethiopia, 2007	Ethiopia	6 months–18 years	<11·0	Admission criteria for therapeutic feeding	5
Bahwere, 2017	Syria	10–14	<16·0	Admission criteria for therapeutic feeding	6
		15–17	<20·0		
		≥18	<22·0		
Martin *et al*., 2009	Western Australia	12–17	<20·0	For initiation for special nutrition care	7
MoHFW, 2017	India	10–18	<16·0	For nutrition support	8
FANTA, 2018	From a sample of countries	10–14	< 16·0	<16·0 cm: SAM	9
				≥16·0 to <18·5 cm: MAM	
				≥18·5 cm: normal	
FANTA, 2018	DRC	10–14	<16·0	For detecting SAM	9
FANTA, 2018	Malawi	10–11	<16·0	For detecting SAM	9
		12–14	<16·0		
		15–18	<18·5		
FANTA, 2018	Mozambique	11–14	<16·0	For detecting SAM	9
		15–18	<21·0		
FANTA, 2018	Namibia	10–14	<16·0	For detecting SAM	9
FANTA, 2018	Tanzania	10–14	<16·0	For detecting SAM	9
		≥15	<18·5		
FANTA, 2018	Uganda	10–14	<16·0	For detecting SAM	9
		15–17	<18·5		
FANTA, 2018	Zambia	10–14	<16·0	For detecting SAM	9
		15–17	<18·5		

CMAM, community management of acute malnutrition; MoHFW, Ministry of Health and Family Welfare, FANTA, Food and Nutrition Technical Assistance Project; DRC, Democratic Republic of Congo; SAM, severe acute malnutrition; MAM, moderate acute malnutrition.

According to the WHO’s IMAI (Integrated Management of Adolescent and Adult Illness) hospital care for adolescents and adults guidelines for the management of illnesses with limited resources, adolescents can be classified as having severe malnutrition (‘severe undernutrition’) if they have MUAC < 160 mm or MUAC = 161–185 mm plus one of the following: pitting oedema up to the knees on both sides, or cannot stand, or sunken eyes^(^^2^^)^. Several countries have adopted their country-specific cut-offs (Table 1). India’s National Nutrition Support for Tuberculosis programme uses an MUAC cut-off of <160 mm for classifying severe thinness in adolescents and determining those eligible for inpatient nutrition rehabilitation and support^(^^8^^)^.

India is home to approximately 120 million adolescent girls, or about 20 % of the world’s population of adolescent girls aged 10–19 years^(^^10^^)^. According to an analysis of a 2015–16 nationally representative survey, 10·6 % of Indian unmarried girls aged 15–19 years are thin (BAZ < −2) and 1·8 % are severely thin (BAZ < −3; International Institute for Population Sciences and UNICEF India, unpublished results). India’s national health programmes have provision for routine nutrition screening for adolescents through utilizing schools and outreach adolescent village health days^(^^11^^)^. The WHO (2007) BAZ charts^(^^1^^)^ have not been adopted in adolescent health programmes. The nutrition assessment is conducted annually by public health workers, using clinical signs, biochemical indicators (Hb) and anthropometry (weight, height). In resource-poor field settings, availability of standardized well-calibrated equipment to measure weight and height and calculation of BMI/BAZ by field workers in the absence of field charts and/or calculators are often challenging. Once identified as severely thin, at present, there is also no policy for provision of nutrition support owing to a lack of dialogue on types and modalities of nutrition support to severely thin adolescents in school/community settings. Use of MUAC for screening severely thin adolescents followed by medical nutrition therapy is restricted to hospital settings in tuberculosis wards only^(^^8^^)^.

In order to use MUAC as a field-friendly proxy for BAZ, there is a need to assess its diagnostic accuracy compared with BAZ, ascertain whether this diagnostic accuracy differs by age or age bracket (early or late adolescents) and initiate a dialogue on modalities and opportunities to provide nutrition support to those severely thin adolescents, as mere screening will not serve the purpose if an intervention is not in place.

We found four India-based studies that compared MUAC measurements in adolescents aged 10–19 years with BMI/BAZ as gold standard (Table 2)^(^^12^^–^^15^^)^, which showed mean MUAC and mean BMI have a correlation (*r*) of 0·35–0·822 (*P* < 0·001). Out of four studies, one study that calculated BAZ reported that MUAC < 18·5 cm and MUAC < 16 cm were in agreement with BAZ of <−2 and <−3, respectively^(^^15^^)^. However, none of these studies were in community settings or identified year-wise MUAC cut-offs for thinness and severe thinness for Indian adolescent girls aged 10–19 years.

**Table 2:** Studies on the correlation between mid-upper arm circumference (MUAC) and BMI/BMI-for-age Z-score (BAZ) in India

Author, year	Location	Age (years)	Sample size	Results	Reference
Dasgupta *et al*., 2010	Kolkata	10–19	194	Burden of thinness: 60·30 % (MUAC < 5th percentile) and 47·9 % (BMI < 5th percentile).	12
				Strong correlation between measurements of MUAC and BMI (*r* = 0·822, se = 0·035, 95 % CI 0·8045, 0·8395, *P* = 0·000000, *r* ^2^ = 0·74). MUAC as a marker was 94·6 % sensitive and 71·2 % specific	
De Kankana, 2016	Paschim Medinipur	10–19	1009	Burden of thinness: 40 % (MUAC) and 24 % (BMI)	13
				BMI and MUAC showed significant correlation (*r* = 0·350, *P* = 0·000)	
				MUAC < 22·9 cm showed: SN = 53·4 %, SP = 79·9 %, PPV = 80·0 % and NPV = 53·6 %	
Jeyakumar *et al*., 2013	Pune, Maharashtra	16–18	565	Burden of thinness: 5·0 % (MUAC < 5th percentile) and 4·8 % (BMI < 5th percentile)	14
				BMI highly correlated with MUAC (*r* = 0·593)	
				MUAC as a screening tool showed SN = 28·57 % and SP = 96·46 %	
Gupta *et al*., 2016	Delhi and Haryana	10–19	4183	Power of association (*r*) between MUAC (cm) and BAZ was 0·68 (*P* < 0·001). MUAC < 18·5 cm was in agreement with BAZ < −2 (*κ* = 0·34; 95 % CI 0·31, 0·38). With BAZ < −2 as gold standard, SN and SP of MUAC < 18·5 cm was 73 and 79 %, respectively	15
				MUAC < 16 cm was compared with BAZ < −3 as gold standard and showed agreement (*κ* = 0·38; 95 % CI 0·31, 0·38). SN and SP of MUAC < 16 cm was 62·6 and 97·3 %, respectively	

SN, sensitivity; SP, specificity; PPV, positive predictive value; NPV, negative predictive value.

Thus, the present study was conducted in two eastern India states (Chhattisgarh and Odisha) to assess the diagnostic accuracy of MUAC compared with BAZ and to provide MUAC cut-offs for screening thinness and severe thinness in adolescent girls, by year and age group (10–14 and 15–19 years). We consider this as a first step towards opening a dialogue on the need for simplified year-wise MUAC field charts for screening of severe thinness in Indian adolescents and thereafter arriving at protocols for management of severe thinness in Indian adolescents as part of adolescent health programmes.

## Methodology

### Data collection

We conducted a cross-sectional study on adolescent girls aged 10–19 years in tribal-dominated districts of Odisha (Koraput and Angul districts) and Chhattisgarh (Bastar district) between October 2016 and April 2017. Overall, in these states, ~8 % of adolescent girls aged 15–19 years have BAZ < −2 (7·8 % in Chhattisgarh and 7·7 % in Odisha) as per an analysis of the Fourth Round of the National Family Health Survey (International Institute for Population Sciences and UNICEF India, unpublished results). Our cross-sectional study was a part of a baseline survey for the evaluation of *Swabhimaan* (meaning ‘self-respect’ or ‘self-pride’), an integrated multisectoral strategy to improve girls’ and women’s nutrition before conception, during pregnancy and after birth. *Swabhimaan* is a collaboration between UNICEF and the State Rural Livelihood Missions of Chhattisgarh and Odisha. A description of the *Swabhimaan* strategy and its impact evaluation design is available elsewhere^(^^16^^)^.

The location and sample size for our cross-sectional study were guided by the design of the *Swabhimaan* strategy, outcome indicators and the change envisaged. Location was four tribal-dominated blocks: Bastar and Bakawand (Bastar district), Pallara (Angul district) and Koraput Sadar (Koraput district). The eligible participants were non-married and non-pregnant adolescent girls aged 10–19 years residing in the study areas. The sample size of eligible participants to be covered was estimated at 3256 adolescent girls (2196 in Chhattisgarh and 1060 in Odisha). Temporary residents, i.e. those adolescents present in homes at the time of the house-to-house census but who said they would migrate within two months of the census, were excluded. Married and/or pregnant adolescent girls, included in other surveys, were also excluded as all variables considered in the current analysis were not available for them. The target sample was collected using simple random sampling. Although we estimated a sample size of 3256 adolescent girls as eligible participants, we interviewed a total of 4648 eligible participants (2921 in Chhattisgarh and 1727 in Odisha). Of the total of 4648 eligible participants interviewed, fourteen adolescents were not given anthropometric measurements and six adolescents’ BAZ was flagged, therefore 4628 adolescent girls were included in the analysis.

Data collection was carried out by thirty investigators, who were supervised by six supervisors. Paper-based method for data collection was used. Written informed consent was obtained from all participants over 18 years of age. For those under 18 years, written consent was taken from their parent(s) or guardian(s) and verbal consent was also taken from the respondents. Utmost confidentiality of information and anonymity of respondents was ensured to prevent linking to any individual. All interviewers participated in a standardization exercise in which they took repeated measurements of ten adolescents in three teams of ten interviewers each. Each interviewer took two height, weight and MUAC measurements for ten participants. We then compared these with supervisors’ (*n* 6) measurements, as well as within teams. The technical error of measurement^(^^17^^)^ for weight was 0·99 and for height was 0·95. Supervisors conducted back-checks for 10 % of interviews.

Ethical approval was obtained from the Institutional Ethics Committees of the All India Institute of Medical Sciences in Chhattisgarh and Odisha. The impact evaluation has been registered with the Registry for International Development Impact Evaluations (RIDIE-STUDY-ID-58261b2f46876)^(^^16^^)^.

A common interview on sociodemographic and household characteristics was administered to all adolescents’ guardians using a pre-tested, structured, bilingual questionnaire (English and Hindi in Chhattisgarh; English and Odia in Odisha). The adolescent girls’ interviews covered sociodemographics and anthropometric measurements (weight, height and MUAC). Anthropometric measurements were conducted using standard techniques^(^^18^^)^. Weight to the nearest 0·1 kg was recorded using a SECA electronic weighing scale with minimal clothing. Height was taken barefoot to the nearest 0·1 cm using a stadiometer. MUAC was measured to the nearest 0·1 cm with a non-stretchable measuring tape (procured from UNICEF supply department). The tape was placed firmly but gently on the arm to avoid compression of soft tissue. The weighing scales and stadiometer were calibrated on a weekly basis prior to data collection with standard weights (1, 2 and 5 kg) and a metre rod (100 cm). The mean se of measurements for height, weight and MUAC across all the data collection teams were insignificant and ranged between 0·001 and 0·025 (95 % CI −0·004, 0·042; *P* < 0·10). The non-response rate was negligible (Chhattisgarh 0·3 %; Odisha 0·4 %). BAZ was calculated using the WHO reference (Stata macro) and classified as <−3 (severe thinness) and <−2 (thinness)^(^^1^^)^.

### Statistical methods

Primary data were entered in CS-Pro version 4·1. Descriptive statistics were generated using the statistical software package IBM SPSS Statistics version 20. MUAC cut-offs for screening thinness and severe thinness and year-wise MUAC cut-offs, as well as those for younger (10–14 years) and older (15–19 years) adolescents, were determined using BAZ < −2 and BAZ < −3, respectively, as the gold standard.

Diagnostic accuracy of MUAC compared with BAZ was assessed using sensitivity (SN), specificity (SP), negative predictive value (NPV) and positive predictive value (PPV), whose values were calculated using the proportion of true positives (TP), false positives (FP), true negatives (TN) and false negatives (FN) using a 2 × 2 table as shown below:

**Table:** 

Thin according to MUAC cut-offs generated in the paper	Thin according to BAZ	
	Yes	No
Yes	TP	FP
No	FN	TN

SN measures the percentage of true positives (thin/severely thin adolescents) calculated as TP/(TP + FN); SP measures the percentage of true negatives (not thin/severely thin adolescents) calculated as TN/(TN + FP); NPV tells us how likely an adolescent is to not be thin if the test is negative, calculated as TN/(TN + FN); and PPV tells us how likely an adolescent is to be thin if the test is positive, calculated as TP/(TP + FP). FP (%) is calculated as FP/(FP + TP) and FN (%) is calculated as FN/(TN + FN). Unlike SN and SP, the NPV and PPV are largely dependent on disease prevalence in an examined population. Values of SN, SP, PPV and NPV were calculated for MUAC cut-offs points against BAZ < −2 and BAZ < −3.

Receiver-operating characteristic (ROC) curve analysis was undertaken to determine the area under the curve (AUC), along with its 95 % CI, to establish the optimal cut-off values of MUAC to identify thinness and severe thinness. The shape of the ROC curve and the AUC determine how high is the discriminative power of a test. The AUC can have any value between 0 and 1 and it is a good indicator of the goodness of the test. The categories used to summarize accuracy of AUC in ROC curve analysis are as follows: excellent (0·9–1·0), good (0·8–0·9), fair (0·7–0·8), poor (0·6–0·7) and fail (0·5–0·1). A test with AUC ≥ 0·85 is considered an accurate test^(19)^. Although AUC gives an overall picture of the behaviour of a diagnostic test across all cut-off values, there remains a necessity to ascertain the specific cut-off value that could be used for screening and for this purpose Youden’s index (YI) is used^(^^20^^)^. The YI is equivalent to the AUC subtended by a single operating point in the ROC curve^(21)^. We calculated YI by deducting 1 from the sum of the test’s SN and SP expressed not as a percentage but as part of a whole number: (SN + SP) − 1. It is one of the oldest measures for diagnostic accuracy, being used for the evaluation of overall discriminative power of a diagnostic procedure and for comparison of this test with other tests^(^^22^^)^. For a test with poor diagnostic accuracy, YI equals 0, and in a perfect test YI equals 1. The YI was calculated using MedCalc software version 17.9.7. Single-age MUAC cut-offs as well as MUAC cut-offs for the groups of younger (10–14 years) and older adolescents (15–19 years) at BAZ < −2 and BAZ < −3 were determined on the basis of the highest corresponding value of YI.

## Results

The analytical sample comprised 4628 adolescents (2910 were from Chhattisgarh and 1718 from Odisha). Their mean age was 14·26 (sd 2·55) years. Sociodemographic characteristics of the participants are described in Table 3. Only 68 % reported being currently enrolled in school. Nearly all (97·2 %) participants belonged to the Hindu religion. Caste-wise, 94 % of participants were from backward castes.

**Table 3:** Sociodemographic characteristics of the sample of adolescent girls aged 10–19 years (*n* 4628) from two eastern India states (Chhattisgarh and Odisha), October 2016–April 2017

Characteristic					Pooled (*n* 4628)							
					Chhattisgarh (*n* 2920)		Odisha (*n* 1728)				MUAC (cm)		BMI (kg/m^2^)		BAZ	
					*n*	%	*n*	%	*n*	%	Mean	sd	Mean	sd	Mean	sd
Age (years)												
10	186	6·4	147	8·6	333	7·2	18·7	2·42	15·3	2·98	−1·0	1·28
11	307	10·6	181	10·5	488	10·5	19·2	2·19	15·4	2·09	−1·1	1·14
12	355	12·2	179	10·4	534	11·5	20·3	2·34	16·1	2·12	−1·1	1·11
13	371	12·8	175	10·2	546	11·8	21·5	2·67	17·1	2·16	−0·9	1·06
14	394	13·5	197	11·5	591	12·8	22·3	2·25	17·8	2·33	−0·9	1·04
15	325	11·2	190	11·1	515	11·1	22·9	2·12	18·2	2·07	−0·9	0·91
16	323	11·1	193	11·2	516	11·2	23·5	2·09	18·8	2·12	−0·8	0·85
17	308	10·6	196	11·4	504	10·9	23·6	2·23	18·7	2·01	−1·0	0·88
18	294	10·1	178	10·4	472	10·2	23·8	1·98	18·9	2·06	−0·9	0·81
19	47	1·6	82	4·8	129	2·8	23·9	2·36	18·6	2·15	−1·1	0·90
Age group (years)												
10–14	1613	55·4	879	51·2	2492	53·9	20·6	2·71	16·5	2·49	−1·0	1·12
15–19	1297	44·6	839	48·8	2136	46·2	23·5	2·15	18·6	2·09	−0·9	0·87
Religion												
Hindu	2859	98·3	1637	95·3	4496	97·2	–		–		–	
Non-Hindu	51	1·8	81	4·7	132	2·9	–		–		–	
Caste												
Scheduled caste	70	2·4	266	15·5	336	7·3	–		–		–	
Scheduled tribe	1895	65·1	928	54·0	2823	61·0	–		–		–	
Other backward caste	811	27·9	389	22·6	1200	25·9	–		–		–	
General	134	4·6	135	7·9	269	5·8	–		–		–	
Currently attending school												
Yes	2179	74·9	2179	74·9	3165	68·4	–		–		–	
No	688	23·6	688	23·6	1267	27·4	–		–		–	
Never gone to school	43	1·5	43	1·5	196	4·2	–		–		–	

MUAC, mid-upper arm circumference; BAZ, BMI-for-age *Z*-score.

Mean BMI of the adolescent girls aged 10–14 and 15–19 years was 16·5 (sd 2·5) and 18·6 (sd 2·1) kg/m^2^, respectively. Corresponding figures for mean BAZ were −1·0 (sd 1·12) and −0·9 (sd 0·87), and those for MUAC were 20·6 (sd 2·7) cm and 23·5 (sd 2·2) cm.

### Correlation between BMI and mid-upper arm circumference

**Fig. 1:**
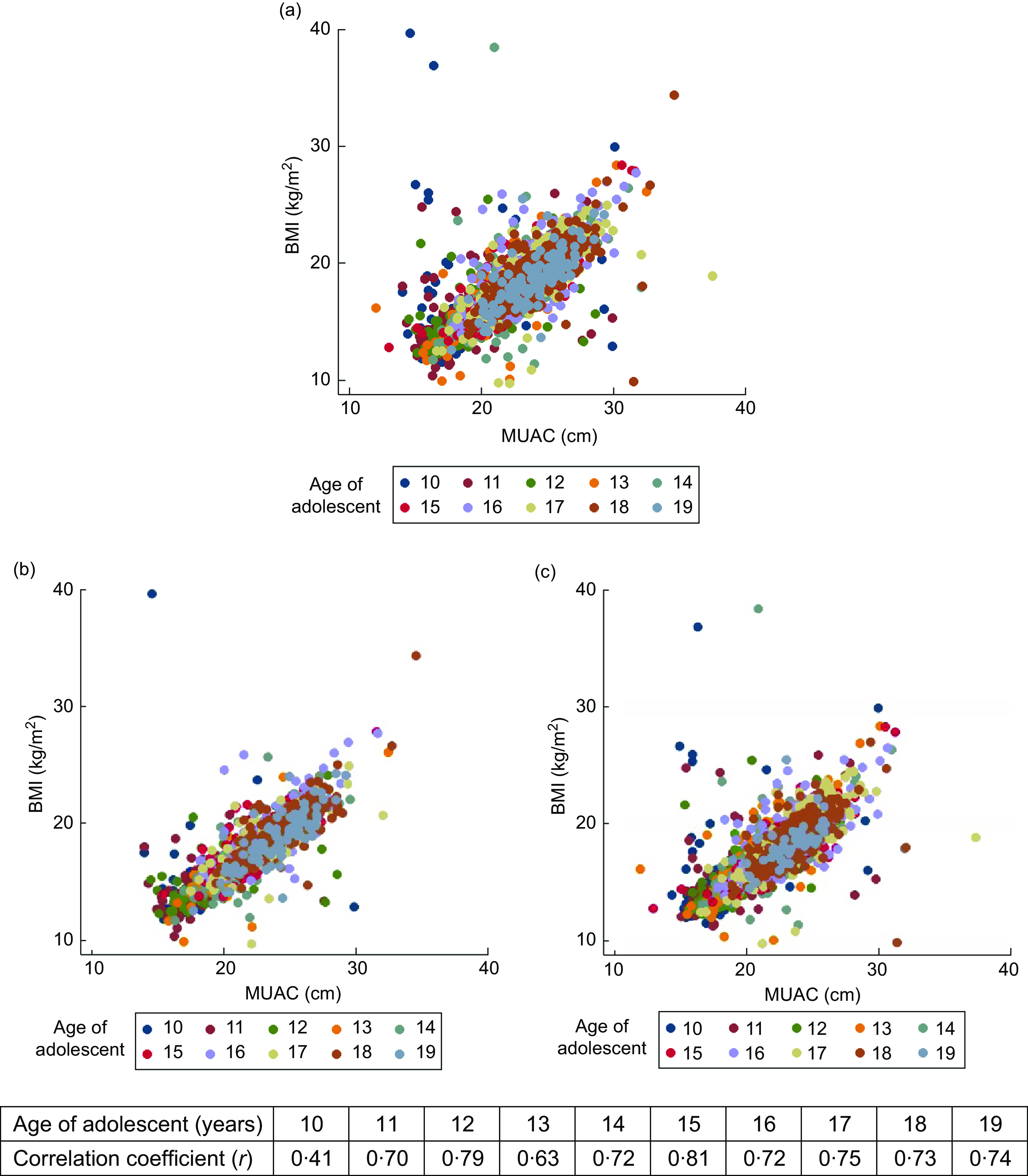
Scatter plots showing the correlation between BMI and mid-upper arm circumference (MUAC), overall and by state, in adolescent girls aged 10–19 years (*n* 4628) from two eastern India states, October 2016–April 2017: (a) pooled (correlation coefficient (*r*) = 0·78, *P* = 0·001); (b) Chhattisgarh (*r* = 0·82, *P* = 0·001); (c) Odisha (*r* = 0·77, *P* = 0·001)

Figure 1 shows the correlation between BMI and MUAC in the pooled data as well as for Chhattisgarh and Odisha, separately. A significant positive correlation was found between measurements of MUAC and BMI (*r* = 0·78, *P* = 0·001, *r*^2^ = 0·61), whereas in state-wise correlation, higher correlation was obtained for Chhattisgarh (*r* = 0·82, *P* = 0·001) than for Odisha (*r* = 0·77, *P* = 0·001). In the age-wise correlation, the lowest correlation was found at 10 years of age (*r* = 0·41, *P* = 0·001) and the highest at 15 years (*r* = 0·81, *P* = 0·001), with all other correlations lying between 0·63 and 0·79 (*P* = 0·001).

**Fig. 2:**
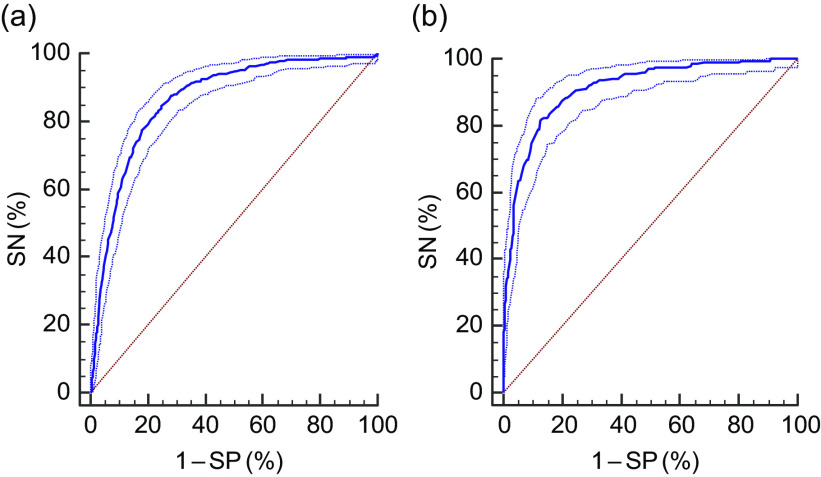
Receiver-operating characteristic curves (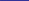) of mid-upper arm circumference to identify thinness (BMI *Z*-score < −2), by age group, among adolescent girls aged 10–19 years (*n* 4628) from two eastern India states (Chhattisgarh and Odisha), October 2016–April 2017: (a) 10–14 years (sensitivity (SN) = 84·5 %; specificity (SP) = 75·1 %; criterion = ≤19·45 cm; area under the curve (AUC) = 0·863; *P* < 0·001); (b) 15–19 years (SN = 82·0 %; SP = 87·0 %; criterion = ≤21·65 cm; AUC = 0·911; *P* < 0·001). (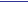) represent the 95 % CI and (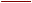) represents the line of no discrimination

**Fig. 3:**
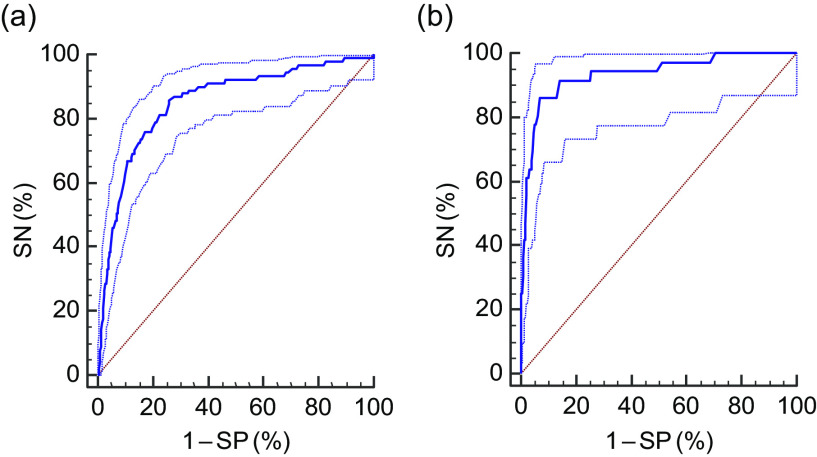
Receiver-operating characteristic curves (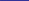) of mid-upper arm circumference to identify severe thinness (BMI *Z*-score < −3), by age group, among adolescent girls aged 10–19 years (*n* 4628) in two eastern India states (Chhattisgarh and Odisha), October 2016–April 2017: (a) 10–14 years (sensitivity (SN) = 85·6 %; specificity (SP) = 74·1 %; criterion = ≤18·95 cm; area under the curve (AUC) = 0·860; *P* < 0·001); (b) 15–19 years (SN = 86·1 %; SP = 93·1 %; criterion = ≤20·70 cm; AUC = 0·934; *P* < 0·001). (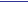) represent the 95 % CI and (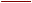) represents the line of no discrimination

### Diagnostic accuracy of mid-upper arm circumference cut-offs for thinness and severe thinness

Table 4 summarizes the MUAC cut-offs for BAZ < −2. The optimal MUAC cut-off to detect thinness among girls aged 10–14 years was ≤19·4 cm (SN = 84·0 %, SP = 75·4 %) and among older adolescents it was ≤21·6 cm (SN = 81·4 %, SP = 87·1 %). Single-age MUAC cut-offs in adolescent girls ranged between 17·7 cm (10 years) and 22·5 cm (19 years) for identifying thinness (BAZ < −2). Overall, the optimal MUAC cut-off for screening thinness among adolescent girls aged 10–19 years was ≤20·9 cm (YI = 0·56, SN = 83·3–86·1 %, SP = 70·3–72·8 %, AUC = 0·85–0·86, *P* = 0·001). Comparing state-wise, the optimal MUAC cut-off for screening thinness among adolescent girls aged 10–19 years was ≤20·9 cm in Chhattisgarh and ≤21·3 cm in Odisha (data not shown). The SN and SP of all the single-age MUAC cut-offs ranged from 70 to 90 % depending on the true positives and true negatives that the age-specific cut-offs could identify. The AUC ranged from 0·84 to 0·94 (*P* = 0·001), signifying good/excellent diagnostic power of the identified single-age cut-offs. At all single-age MUAC cut-offs, the NPV was much higher than the PPV, signifying that the MUAC cut-offs were able to correctly exclude adolescents without thinness. We also did an additional analysis where we obtained the single-age and grouped cut-offs for moderate thinness (BAZ < −2 and ≥−3; data not shown). There was no significant difference obtained in the cut-offs for thinness and moderate thinness.

**Table 4:** Diagnostic test accuracy measures for varying cut-offs of mid-upper arm circumference (MUAC) for predicting thinness (BMI-for-age *Z*-score < −2 as gold standard) among adolescent girls aged 10–19 years (*n* 4628) from two eastern India states (Chhattisgarh and Odisha), October 2016–April 2017

Age (years)	MUAC cut-off (cm)	SN (%)	SP (%)	YI	FN (%)	FP (%)	PPV (%)	NPV (%)	AUC	95 % CI
10	≤17·7	87·3	71·1	0·58	4·0	58·6	41·4	96·0	0·84	0·79, 0·87
11	≤18·1	81·6	81·2	0·62	5·4	48·0	52·0	94·6	0·87	0·84, 0·90
12	≤19·0	87·3	86·0	0·73	4·1	35·8	64·2	95·9	0·92	0·89, 0·94
13	≤20·1	90·0	82·4	0·72	1·7	57·2	42·8	98·3	0·92	0·89, 0·94
14	≤20·6	77·9	85·4	0·63	3·7	55·5	44·5	96·2	0·89	0·87, 0·92
15	≤20·8	71·6	93·9	0·65	3·3	42·5	57·5	96·6	0·92	0·89, 0·94
16	≤21·6	79·4	88·0	0·67	1·8	64·7	35·3	98·1	0·91	0·89, 0·94
17	≤21·7	91·3	88·2	0·79	0·9	56·3	43·7	99·1	0·94	0·92, 0·96
18	≤22·3	83·6	81·3	0·65	2·2	65·8	34·1	97·8	0·89	0·86, 0·91
19	≤22·5	72·2	82·3	0·54	5·2	60·7	39·3	94·8	0·86	0·79, 0·92
10–14	≤19·4	84·0	75·4	0·59	4·2	58·7	41·3	95·8	0·86	0·84, 0·87
15–19	≤21·6	81·4	87·1	0·68	2·2	60·0	40·0	97·8	0·91	0·89, 0·92

SN, sensitivity; SP, specificity; YI, Youden index; FN, false negative; FP, false positive; PPV, positive predictive value; NPV, negative predictive value; AUC, area under the (receiver-operating characteristic) curve.

Table 5 summarizes the MUAC cut-offs for BAZ < −3. Single-age MUAC cut-offs in adolescent girls ranged between 17·0 cm (10 years) and 21·5 cm (19 years) for severe thinness (BAZ < −3). The optimal MUAC cut-off to detect severe thinness among girls aged 10–14 years was ≤18·9 cm (SN = 84·6 %, SP = 74·1 %) and among older adolescents it was ≤20·7 cm (SN = 86·1 %, SP = 93·1 %). The SN and SP of the single-age MUAC cut-offs ranged from 70 to 100 %. The AUC were in the range of 0·84–0·97, signifying good/excellent diagnostic power of the identified single-age cut-offs. The PPV of the single-age MUAC cut-offs ranged from 8 to 30 %, while the NPV was >99 % for all ages. Thus, the PPV was lower for single-age MUAC cut-offs with BAZ < −3 as the gold standard compared with MUAC cut-offs with BAZ < −2, while the NPV was higher. The optimal MUAC cut-off for screening severe thinness among adolescent girls aged 10–19 years was ≤19·5 cm (YI = 0·52–0·69, SN = 0·63–87·7 %, SP = 81·3–88·9 %, AUC = 0·83–0·90, *P* = 0·001). Comparing state-wise, the optimal MUAC cut-off for screening severe thinness among adolescent girls aged 10–19 years was ≤19·4 cm in Chhattisgarh and ≤18·5 cm in Odisha (data not shown).

**Table 5:** Diagnostic test accuracy measures for varying cut-offs of mid-upper arm circumference (MUAC) for predicting severe thinness (BMI-for-age *Z*-score < −3 as gold standard) among adolescent girls aged 10–19 years (*n* 4628) from two eastern India states (Chhattisgarh and Odisha), October 2016–April 2017

Age (years)	MUAC cut-off (cm)	SN (%)	SP (%)	YI	FN (%)	FP (%)	PPV (%)	NPV (%)	AUC	95 % CI
10	≤17·0	87·5	79·6	0·67	0·3	90·4	9·6	99·7	0·87	0·83, 0·91
11	≤17·6	94·7	79·3	0·74	0·2	84·4	15·6	99·8	0·90	0·87, 0·92
12	≤18·2	91·3	84·5	0·75	0·4	79·0	21·0	99·6	0·94	0·91, 0·96
13	≤18·8	85·0	92·8	0·77	0·6	69·0	31·0	99·4	0·91	0·88, 0·93
14	≤20·3	71·4	84·6	0·56	1·2	85·4	14·6	98·8	0·84	0·81, 0·87
15	≤20·3	100·0	93·4	0·93	0·0	71·7	28·3	100·0	0·97	0·95, 0·98
16	NA*	NA*	NA*	NA*	NA*	NA*	NA*	NA*	NA*	NA*
17	≤20·7	76·9	94·1	0·71	0·6	74·3	25·7	99·4	0·91	0·89, 0·94
18	≤21·1	75·0	92·5	0·67	0·2	92·1	7·9	99·8	0·97	0·95, 0·98
19	≤21·5	80·0	87·3	0·67	0·9	80·0	20·0	99·1	0·84	0·76, 0·90
10–14	≤18·9	84·6	74·1	0·58	0·7	89·1	10·9	99·3	0·86	0·83, 0·86
15–19	≤20·7	86·1	93·1	0·79	0·2	82·2	17·8	99·8	0·93	0·92, 0·94

SN, sensitivity; SP, specificity; YI, Youden index; FN, false negative; FP, false positive; PPV, positive predictive value; NPV, negative predictive value; AUC, area under the (receiver-operating characteristic) curve.

* NA = cases insufficient to estimate a reliable MUAC cut-off for severe thinness.

Each ROC curve shows the trade-off between sensitivity and specificity. The AUC for thinness among girls aged 10–14 and 15–19 years was 0·86 and 0·91, respectively (*P* < 0·0001; Fig. 2). Similarly, the AUC for severe thinness among girls aged 10–14 and 15–19 years was 0·86 and 0·93, respectively (*P* < 0·0001; Fig. 3). The AUC values were highly significant and the curves were closer to top left corner, indicating high accuracy of the test to detect thinness and severe thinness.

### Prevalence of thinness and severe thinness

The burden of thinness (BMI < −2) and severe thinness (BMI < −3) among adolescent girls aged 10–14 years was 17·1 and 3·6 %, respectively. Corresponding figures for adolescent girls aged 15–19 years were 9·6 and 1·7 % (Table 6). By BAZ, the prevalence of thinness and severe thinness was highest among adolescents aged 12 years which counted for 22·6 and 4·5 %, respectively; while the lowest prevalence was found in age 16 years where 7·5 % were thin. According to MUAC, the burden of thinness among adolescent girls aged 10–14 years (MUAC ≤ 19·4 cm) and 15–19 years (MUAC ≤ 21·6 cm) was 41·3 and 40·0 %, respectively; whereas the burden of severe thinness among adolescent girls aged 10–14 years (MUAC ≤ 18·9 cm) and 15–19 years (MUAC ≤ 20·7 cm) was 11·0 and 17·7 %, respectively. The burden of thinness and severe thinness was higher when assessed with MUAC compared with BAZ in both age groups (Table 6).

**Table 6:** The burden of thinness and severe thinness based on mid-upper arm circumference (MUAC) and BMI-for-age *Z*-score (BAZ) among adolescent girls aged 10–19 years (*n* 4628) from two eastern India states (Chhattisgarh and Odisha), October 2016–April 2017

	Thinness (BAZ < −2)			Severe thinness (BAZ < −3)		
Age (years)	MUAC cut-off (cm)	MUAC-based prevalence (%)	BAZ-based prevalence (%)	MUAC cut-off (cm)	MUAC-based prevalence (%)	BAZ-based prevalence (%)
10	≤17·7	41·3	19·2	≤17·0	9·6	2·4
11	≤18·1	52·8	20·3	≤17·6	16·4	4·1
12	≤19·0	66·2	22·6	≤18·2	22·0	4·5
13	≤20·1	43·0	12·9	≤18·8	29·3	3·6
14	≤20·6	44·4	13·3	≤20·3	15·0	3·5
15	≤20·8	57·5	10·3	≤20·3	30·4	2·9
16	≤21·6	35·1	7·5	NA*	NA†	0·2
17	≤21·7	47·8	9·5	≤20·7	11·5	2·6
18	≤22·3	34·2	10·6	≤21·1	7·5	0·8
19	≤22·5	65·0	13·7	≤21·5	20·0	3·8
10–14	≤19·4	41·3	17·1	≤18·9	11·0	3·6
15–19	≤21·6	40·0	9·6	≤20·7	17·7	1·7

* NA = cases insufficient to estimate a reliable MUAC cut-off for severe thinness.

† NA = cases insufficient to estimate a reliable MUAC cut-off for severe thinness. Hence, prevalence cannot be estimated.

## Discussion

The present study has several important inferences pertaining to prevalence of thinness and severe thinness in adolescents aged 10–19 years using BAZ and MUAC as well as year-wise MUAC cut-offs for screening thinness and severe thinness.

First, the burden of thinness (BAZ < −2) among adolescent girls using identified the MUAC cut-off was fourfold higher compared with BAZ (e.g. MUAC-based 40 %, BAZ-based 9·6 %, at 15–19 years). There is limited information from India on use of MUAC for detection of thinness among adolescent girls. Community-based studies reported a burden of thinness among adolescent girls between 24 and 48 % (according to BMI) and between 40 and 60 % (according to MUAC)^(^^12^^,^^13^^)^. However, one study reported lower burden of thinness as 4·8 % (BMI) and 5·0 % (MUAC), respectively^(^^16^^)^.

Second, a significant correlation was found between BAZ and MUAC measurements (*r* = 0·78, *P* = 0·001, *r*^2^ = 0·61). Our study results are comparable with all three studies which also reported a significant correlation between BMI and MUAC measurements^(^^12^^,^^13^^,^^16^^)^. The study by Dasgupta *et al*. documented that MUAC is highly sensitive (97 %) and specific (71 %) in the screening of malnourishment among adolescents (10–19 years)^(^^13^^)^. De Kankana^(^^13^^)^ found that the mean MUAC was 21·7 cm and implied that BMI and MUAC have higher and significant correlation. MUAC can be a useful and efficient index for the screening of thinness, generally assessed from BMI. Gupta *et al*.^(^^15^^)^ showed that the power of association between MUAC and BAZ < −2 was considerably high, and that MUAC can be a gold standard in assessment of thinness. They also found that MUAC < 18·5 cm was in agreement with BAZ < −2 and MUAC cut-off of <16·5 cm with BAZ < −3. A survey conducted among 565 adolescent girls (16–18 years) from Pune, Maharashtra, warranted that MUAC had high specificity but low level of sensitivity^(^^14^^)^.

Third, our results show that the MUAC cut-off to detect thinness and severe thinness in young adolescent girls (10–14 years) was ≤19·4 and ≤18·9 cm, respectively, and it was ≤21·6 and ≤20·7 cm for late adolescent girls (15–19 years). The specificity of the MUAC cut-off (≤20·7 cm) among 15–19-year-old adolescents for BAZ < −3 (93·1 %) was higher than the specificity of the MUAC cut-off (≤21·6 cm) for BAZ < −2 (87·1 %), signifying that MUAC can be more specific for diagnosis of thinness in a severely malnourished population. For both BAZ < −2 and BAZ < −3, the specificity was higher for older adolescents (15–19 years) compared with younger adolescents (10–14 years; 75·4 and 87·1 % for BAZ < −2; 74·1 and 93·1 % for BAZ < −3), with sensitivity being similar for both. Hence, the MUAC cut-off is more specific for diagnosis of thinness in older adolescents, probably because they are fully grown, so there is less variation in the cut-offs. The results of the present study were corroborated by previous studies. Gupta *et al.* ^(^^15^^)^ found that with BAZ < −2 as the gold standard, MUAC cut-off of <18·5 cm had a sensitivity and specificity of 73 and 79 %, respectively, for detecting thinness in 10–19-year-old adolescent girls. In the same study, higher specificity (97·3 %) was obtained with BAZ < −3 as the gold standard and MUAC cut-off of <16 cm^(^^15^^)^. Another study reported similar findings on 10–19-year-old adolescent girls, with MUAC cut-off of <22·9 cm showing a sensitivity of 53·4 % and a much higher specificity of 79·9 %^(^^13^^)^. A survey conducted with 565 adolescent girls (16–18 years) from Pune, Maharashtra, also warranted that MUAC had high specificity (96·5 %) but low level of sensitivity (28·5 %)^(^^14^^)^. Thus, results indicate that MUAC has higher specificity and lower sensitivity, particularly for detecting thinness below the BAZ cut-off of −3. It is, therefore, important to relate MUAC to age and sexual maturity of individual girls for a meaningful identification of thinness^(^^23^^)^, which was missed in the present study. From a programmatic perspective, it is not feasible to define a single MUAC cut-off to identify nutritionally at-risk adolescent girls between the ages of 10 and 19 years.

Fourth, we found that with BAZ < −2 as the gold standard, the NPV was much higher than the PPV at all single-age MUAC cut-offs (PPV = 35–65 %, NPV > 95 %) signifying that the MUAC cut-offs were able to correctly exclude adolescents without thinness. Similar findings (NPV (>99 %) > PPV (10–30 %) at all cut-offs) were obtained with BAZ < −3 as the gold standard. Thus, MUAC cut-offs were able to correctly exclude adolescents who were not thin according to BAZ. We also found that PPV was lower at MUAC cut-offs with BAZ < −3 as the gold standard compared with MUAC cut-offs with BAZ < −2, while the NPV was higher. This is because, unlike sensitivity and specificity, predictive values are largely dependent on disease prevalence in the examined population. The prevalence of thinness would be much higher with the BAZ < −2 cut-off than with the BAZ < −3 cut-off^(^^17^^)^. Hence the PPV was much higher at BAZ < −2 than at BAZ < −3. However, PPV and NPV from one study should not be transferred to some other setting with a different prevalence of the disease in the population. Hence it is better to use SN and SP indicators for comparing results across different populations. There was no difference in the MUAC cut-offs, SN and SP values before and after adjusting for the outliers in the data.

Although our study identified both single-age MUAC cut-offs and cut-offs for younger (10–14 years) and older (15–19 years) adolescent girls, it is preferable to use single-age MUAC cut-offs due to wide variations in the cut-offs. For instance, the optimal MUAC cut-off to detect thinness (BAZ < −2) among adolescent girls aged 10–14 years was found to be ≤19·4 cm, while the cut-off at 10 and 14 years was ≤17·7 and ≤20·6 cm, respectively, having a wide variation of 2·9 cm. Thus, the chances of classifying a 10-year-old adolescent as thin is higher using the cut-off of 19·4 cm as compared with the single-age cut-off of 17·7 cm. Hence, it is important that single-age MUAC cut-offs are used in field settings for identifying thinness among adolescents. However, in cases where the age of the adolescent is not known, the grouped cut-offs for younger and older adolescents can be used: MUAC ≤ 19·4 cm (10–14 years) and MUAC ≤ 21·6 cm (15–19 years). There are other considerations about what should be the nutrition support provided to adolescent girls with severe thinness, such as an extra meal or linkage with social protection, for which the assessment measure may be used to determine the response plan. This was outside the scope of the present paper, however it requires deliberation.

While MUAC is particularly useful in remote areas, where it is not possible to carry the weighing machine or stadiometer over long distances and so calculation of BMI/BAZ is not feasible (hence MUAC tapes become handy in such places), there are some aspects of MUAC that should be kept in mind. First, MUAC changes substantially with age during adolescence, especially at the younger ages when growth patterns and physical maturity differ largely between individual girls. As a result, different cut-offs must be used for adolescents of different ages. This requires an accurate age for each survey subject in order to judge whether she falls above or below an age-specific cut-off. Second, despite the convenience and ease of measurement of MUAC, it requires careful training and supervision in order to prevent wrapping the measuring tape too tightly or too loosely, which results in an erroneous estimate and some degree of observer variability.

### Strengths and limitations

The present community-based study was conducted on a reasonable sample size with good quality control and monitoring. The sample was drawn systematically from rural deprived areas where programmes and interventions to improve nutrition are intended. However, the following limitations merit consideration. Unlike previous studies, the current study has not defined one single MUAC cut-off to identify nutritionally at-risk adolescent girls between the ages of 10 and 19 years; rather single-age MUAC cut-offs were established for detection of thinness among adolescent girls. The study falls short in evaluating any health consequences and, therefore, cannot compare one method over another. The sample being selected from only two geographical areas is another limiting factor of the study, limiting generalizability of the results. Since we found differences in MUAC between the two states, we need large-scale data to arrive at national year-wise cut-offs for MUAC in Indian adolescents (both girls and boys) for appropriate interventions in emergency situations, field settings and outpatient therapeutic clinics as MUAC field charts. Moreover, since the sample was majorly drawn from poverty pockets in India, the analysis cannot be used to derive at MUAC cut-offs for overweight and obesity. Finally, the present study did not cover adolescent boys aged 10–19 years.

## Conclusion

To conclude, MUAC cut-off points with good predictive ability to detect thinness among adolescent girls aged 10–14 years (young adolescents) and 15–19 years (late adolescents) were ≤19·4 and ≤21·6 cm, respectively. The age-wise, sex-wise and context-specific MUAC cut-offs should be preferred in place of one MUAC cut-off across 10–19 years, based upon several considerations including purpose, burden and logistic resources. Availability of MUAC field charts by year, for adolescent boys and girls, by context/region, will prove useful in settings where BAZ/BMI is not available. However, prior evidence from large-scale representative survey data wherein MUAC and BAZ measurements have been taken for adolescents is needed to prove/disprove MUAC diagnostic accuracy and suitability for the specific region/context compared with BAZ.
